# The effect of a monetary incentive for administrative assistants on the survey response rate: a randomized controlled trial

**DOI:** 10.1186/s12874-016-0201-8

**Published:** 2016-08-05

**Authors:** Arnav Agarwal, Dany Raad, Victor Kairouz, John Fudyma, Anne B. Curtis, Holger J. Schünemann, Elie A. Akl

**Affiliations:** 1 Department of Clinical Epidemiology and Biostatistics, McMaster University, CRL-209, 1280 Main St. West, Hamilton, ON L8S 4K1 Canada; 2 Faculty of Medicine, University of Toronto, 1 King’s College Circle #3172, Toronto, ON M5S 1A8 Canada; 3 Department of Medicine, State University of New York at Buffalo, 3435 Main Street, 14214 Buffalo, NY USA; 4 Department of Medicine, McMaster University, 1280 Main St. West, Hamilton, ON L8S 4K1 Canada; 5 Department of Internal Medicine, American University of Beirut, Riad El Solh, PO Box 11-0236, Beirut, 1107 2020 Lebanon

## Abstract

**Background:**

There is sufficient evidence that monetary incentives are effective in increasing survey response rates in the general population as well as with physicians. The objective of this study was to assess the impact of a monetary incentive intended for administrative assistants on the survey response rate of physicians in leadership positions.

**Methods:**

This was an ancillary study to a national survey of chairs of academic Departments of Medicine in the United States about measuring faculty productivity. We randomized survey participants to receive or not receive a $5 gift card enclosed in the survey package. The cover letter explained that the gift card was intended for the administrative assistants as a “thank you for their time.” We compared the response rates between the 2 study arms using the Chi-square test.

**Results:**

Out of 152 participants to whom survey packages were mailed to, a total of 78 responses were received (51 % response rate). The response rates were 59 % in the incentive arm and 46 % in the no incentive arm. The relative effect of the incentive compared to no monetary incentive was borderline statistically significant (relative risk (RR) = 1.36, 95 % confidence interval (CI) 0.99 to 1.87; *p* = 0.055).

**Conclusion:**

Monetary incentives intended for administrative assistants likely increase the response rate of physicians in leadership positions.

## Background

Surveying physicians using a mailed questionnaire has been associated with low response rates [1–5]. A recent systematic review also found a decline over recent years in survey response rate among clinicians [6]. A low response rate can introduce bias and uncertainty to the survey results [1, 2, 7–10]. Therefore, multiple studies have focused on methods and strategies to increase response rate, particularly for mailed survey questionnaires to physicians. These methods include incentive-based approaches such as small financial incentives and token non-monetary incentives, and design-based approaches primarily focused on mode of survey questionnaire delivery [11].

Monetary incentives can increase response rates to mailed surveys. A recent systematic review including 48 studies and assessing strategies employed in surveys of health professionals found an estimated 12 percentage points increase in response rates with the introduction of monetary incentives [6]. Another systematic review of 66 published studies found that even a small financial incentive was effective in improving physician response rate [11]. A 2010 workshop convened by the National Cancer Institute also recognized the importance of monetary incentives in increasing mailed survey response rates, and noted that incentive amount might influence how effective the given incentive is [12].

A high number of published trials of interventions to increase survey response rates have recruited general or specialty physicians [13–29], but very few have specifically addressed physicians in leadership positions [28, 29]. We hypothesized that small financial incentives (e.g., $5 gift cards) may be unappealing to these physicians, and therefore would likely not increase their response rate. However, targeting their administrative assistants with the monetary incentive might have a greater impact.

Typically, the administrative assistants receive and screen survey requests, conveying those they deem to be of enough importance to the physician [3, 11, 30]. They potentially receive direct directives from the physician to refuse all survey requests, and thus act as the gatekeepers [3]. Providing administrative assistants with a monetary incentive may increase their willingness to bring a survey request to the attention of the physician, and to remind their physicians to complete a survey.

No previous study has examined the impact of monetary incentives targeted towards administrative assistants or other gatekeepers on response rates of physicians in leadership positions [12]. Therefore, we conducted a randomized controlled trial (RCT) to assess the impact of a monetary incentive intended for the administrative assistants on the response rate of physicians in leadership positions.

## Methods

We conducted the RCT as an ancillary study to a national survey of Chairs of academic Departments of Medicine in the United States [31]. The aim of the survey was to evaluate whether and how these academic departments measure faculty productivity for the purpose of salary compensation.

### Trial design

We randomized participants to one of two arms: a) the incentive arm that consisted of a survey package with a $5 Starbucks gift card enclosed; b) the no incentive arm that consisted of a survey package without a gift card enclosed. The cover letter included the following explanation regarding the intended purpose of the enclosed $5 gift card for the incentive arm: “Please feel free to have your assistant or a faculty member fill out the survey. We are enclosing a $5 Starbucks gift card as a thank you for their time.” The cover letter provided to the no incentive arm included only the first of the two statements. We used Microsoft Excel to generate a random sequence of numbers.

### Study population

Our study population consisted of the chairpersons of all academic Departments of Medicine in the United States. We excluded chairpersons of Departments of Medicine based in the Veterans Affairs Healthcare System for reasons related to the subject of the survey (i.e., compensation for academic productivity) [31]. We obtained the names and contact information of the chairpersons from a commercial vendor (Data Services, Inc.). The University at Buffalo Institutional Review Board approved the study and waived the requirement for a signed consent form. Instead, we sent participants a study information sheet that included all information typically included in a consent form.

### Survey design

The survey questionnaire consisted of eight questions with 23 tabulated close-ended items addressing whether and how departments measure faculty productivity for the purpose of salary compensation [32]. Initially, we notified potential participants via the listserv of Chairs of Internal Medicine about the upcoming survey. Then, we mailed participants a survey package including a personalized cover letter, the survey questionnaire, and a pre-addressed stamped return envelope. Two weeks after the initial mailing we sent non-responders a follow-up letter. Two weeks later we attempted to contact non-responders by phone.

We used the following survey methods to maximize response rate [7, 11]: pre-mailing notification, personalized cover letter with university sponsorship, use of colored ink, first class mailing, inclusion of a stamped return envelope, follow-up mail, including a new copy of the questionnaire in the follow-up mail, follow-up phone call, and a short questionnaire focusing on factual questions.

### Statistical analysis

The primary outcome was the response rate, which was defined as the number of completed questionnaires divided by the sample size. We conducted statistical analyses using the Chi-Square test to compare the baseline characteristics of respondents and the final response rates between the two study arms. We considered two-sided p values and 0.05 for statistical significance. Our sample included all potentially eligible chairpersons of Departments of Medicine.

## Results

We included in the survey study a total of 152 chairpersons of Departments of Medicine and their respective administrative assistants. The numbers of responses were: 39 (26 %) to the first mailing, 32 (21 %) to the second mailing, and 7 (5 %) to the follow-up phone call. The total number of responses was 78, yielding an overall response rate of 51 % (Table 1).

**Table 1:** Response rates of incentive and no incentive arms

	Responses/total	Response rate (%)	Relative risk (95 % CI)	*p* value
Incentive arm	45/76	59 %	1.36 (0.99 to 1.87)	0.055
No incentive arm	33/76	46 %		

The response rates were 59 % in the incentive arm and 46 % in the no incentive arm, representing a difference of 13 percentage points in response rate. The relative effect of the incentive arm compared to no incentive arm was relative risk (RR) = 1.36 (95 % Confidence Interval (CI) 0.99 to 1.87; *p* = 0.055) (Table 1).

## Discussion

We conducted a randomized controlled trial to assess the impact of a $5 gift card intended for the administrative assistants on the response rate of chairpersons of the departments of internal medicine in the United States. We found that this monetary incentive likely increased the overall response rate.

While the extent of gatekeeping in physician surveys has not been previously documented, several studies have hypothesized that they put a downward pressure on response rates [11, 30, 33–35]. A National Cancer Institute workshop in 2010 agreed that the intended respondents not likely being the initial points of contact represented a major obstacle to physician survey uptake, and that non-physician staff serve as important gatekeepers in sorting and opening mail and screening telephone calls for physicians [12]. While studies have used different means of targeting physician respondents and their gatekeepers to increase survey response rates, incentives have not targeted gatekeepers directly, and have not been monetary; in general, most studies discussing targeting gatekeepers with incentives have been speculative in nature.

Previous studies have assessed the effects of monetary incentives, although they did not target administrative assistants. Donaldson et al. found a 12 percentage point increase in probability of surveys being returned with the inclusion of a $5 check [17]. Similarly, Asch et al. found a 15 percentage point increase in response rates of American primary care physicians receiving a mailed survey with a $5 bill versus a $2 bill as a monetary incentive [13]. In a mailed survey to medical directors of large medical groups and independent practice associations, Malin et al. found an increase from 17 to 13 % with first and second mailings of a survey with no monetary incentive, to 66 % in a third mailing with a $50 monetary incentive included [28]. This association has been observed in groups others than physicians as well; a recent systematic review of survey response rates found that using monetary incentives doubled postal survey response rates among varied populations including patients, other healthcare providers, and participants in non-health studies [36].

Studies have generally reported that different amounts of monetary incentives are associated with differences in physician response rates. Kasprzyk et al. assessed the effect of varying cash incentives ($0, $15, $25) on 311 physicians and found a 43 percentage point increase in response rate between physicians receiving an incentive and physicians receiving no incentive, with a 7.7 percentage point difference between a $25 incentive and $15 incentive with delivery by first class mail [19]; Berk et al. reported similar findings [14]. Halpern et al. found the inclusion of $10 versus a $5 cash incentive in initial mailings of a questionnaire increased response rates by 7.7 percentage points (*p* = 0.009) as well [18]. Griffin et al. compared the response rates of a $5 monetary incentive relative to a $2 monetary incentive, and found a significantly higher response rate after the first mailing but a non-significant difference after the second mailing [37].

Contributing to this evidence, our findings suggest that those conducting surveys targeting individuals in leadership positions, particularly physicians, may be able to increase response rate by providing a monetary incentive to their administrative assistants. The 13 % increase in response rate with the administration of a small monetary incentive in our study is comparable to the response rate changes reported by Donaldson et al. [17] and Asch et al. [13] with incentives targeting physicians directly, and is greater than the difference in response rates observed between larger and smaller physician-targeted monetary incentives as reported by Kasprzyk et al. [19], Berk et al. [14], Halpern et al. [18] and Griffin et al. [37]. Our findings therefore suggest that targeting the administrative assistants and gatekeepers of physicians in leadership positions may be as effective in increasing survey response rates as monetary incentives targeting physicians directly, and are likely more effective than increasing the monetary amount of these physician-targeted incentives.

To the best of our knowledge, no previous surveys or systematic reviews have addressed the impact of a monetary incentive to administrative assistants in increasing the response rate of individuals in leadership positions. One limitation of our study is the relatively small sample size. Secondly, the generalizability of our results to physicians in other types of leadership positions, and their administrative assistants, may be limited. A third potential limitation is that we included in the cover letter a statement that a fellow faculty member or the chair assistant could complete the survey, but we did not collect information on who completed the questionnaire. This concern is minimized by the fact that the subject of the survey is a technical one (how departments measure faculty productivity) and not addressing personal characteristics (e.g., attitudes, beliefs or knowledge). The results of our study would not apply in the latter case. Irrespective of who completed the questionnaire, our study would still be relevant to the gatekeeper role of the administrative assistant who has at least some control on whether the survey gets passed along or not.

## Conclusions

Monetary incentives intended for administrative assistants likely increase the survey response rate of physicians in leadership positions. They are likely equally effective in increasing response rates as physician-targeted monetary incentives, and may be more effective than increasing the amount of a given physician-targeted monetary incentive. Future trials should target the administrative assistants of individuals with other types of leadership positions in both health and non-healthcare settings. The trials may also compare the effectiveness of different types and different levels of monetary incentives. While this study addresses only one measure of survey quality, i.e., response rate, future studies should also address other measures such as survey design, sample coverage, and validity of the survey tool.

## Abbreviations

CI, confidence interval; RCT, randomized controlled trial; RR, relative risk
